# Editorial: Health informatics in pediatric gastroenterology, hepatology & nutrition

**DOI:** 10.3389/fped.2024.1458319

**Published:** 2024-11-11

**Authors:** Daniele Zama, Steven T. Leach

**Affiliations:** ^1^ Pediatric Emergency Unit, IRCCS Azienda Ospedaliero-Universitaria di Bologna, Bologna, Italy; ^2^ Department of Medical and Surgical Sciences, Alma Mater Studiorum University of Bologna, Bologna, Italy; ^3^ School of Clinical Medicine, UNSW Medicine & Health, Sydney, NSW, Australia

**Keywords:** gastroenterology, hepatology, nutrition, pediatric, informatics

**Editorial on the Research Topic**
Health informatics in pediatric gastroenterology, hepatology & nutrition

The use of big data and information technology, health informatics enables clinicians to collect, analyze, and utilize digital health information. The application of informatics in the fields of health and medicine has the potential to revolutionise patient care in the future. Indeed,

In pediatrics, health information goes beyond the needs of the patient to include the needs of the family and other caregivers. Effective health informatics solutions can help ensure high-quality care for infants and children. This special issue focuses on the novelties coming from health informatics application and promotes important discussions in the Pediatric Gastroenterology, Hepatology & Nutrition area, characterized by diseases that are frequent in this population with a high impact on the quality of life.

Particularly, considering their frequency in the pediatric population these diseases represent a perfect test field for informatic applications that could renew the clinical assistance of these patients.

The review by Zhou et al. delves into irritable bowel syndrome (IBS), enhancing our comprehension of the direct and indirect mechanisms underlying early life-related IBS and providing new insights and research directions from childhood to adulthood. IBS is a widespread functional gastrointestinal disorder globally. Extensive research has uncovered multiple factors contributing to its development, such as genetic predisposition, chronic infection, gut dysbiosis, abnormal serotonin metabolism, and brain dysfunction. Recent studies have highlighted the crucial role of early life as a vulnerability period for IBS. Current evidence indicates that diet can increase the risk of IBS in offspring by affecting microbiota composition and the intestinal epithelium.

Therefore, the authors compile the existing literature and identify the determinants studied to date that impact on the future risk of developing IBS from the earliest stages of life.

The manuscript by Shaaban et al. introduces a previously validated algorithm for the use of the Ketogenic Diet (KD) in managing patients, particularly those with specific types of epilepsy, those experiencing adverse effects, or those unresponsive to pharmacological treatments. It was developed by a multidisciplinary team of experts at the Children's Hospital, Ain Shams University, Egypt. KD, which is characterized by high fat, restricted carbohydrates, and adequate protein, has proven to be an effective nonpharmacological treatment for drug-resistant epilepsy by producing ketones that serve as an alternative fuel source for the brain, thereby reducing seizure frequency. The benefits of this strategy are attributed to its universal availability, various administration methods, and cost-effectiveness. This algorithm serves as a guide for implementing the KD in treating drug-resistant epilepsy in children.

The interesting original research article performed by Ma et al. explored a previously unexamined correlation between 25(OH)D levels, *Helicobacter pylori* colonization, and the degree of inflammation. The authors reported that low gastric acid levels due to HP-related chronic gastritis could reduce the absorption of iron, vitamin B12, other micronutrients, and vitamin D. In addition, the degree of gastric mucosal inflammation and activity in the mucosa was positively correlated with HP colonization. However, serum 25(OH)D levels were negatively correlated with both HP colonization and the degree of inflammation in the gastric mucosa. These findings indicate that vitamin D absorption is affected by HP infection, with the impact increasing as the severity of the infection escalates.

The original research article by Huang et al. evaluated three supervised machine-learning (ML) methods—support vector machine, random forest, and light gradient boosting machine (LGBM). The goal of the study was to classify children with intractable functional constipation (IFC) into three subtypes based on symptom severity, self-efficacy, quality of life (measured using certified questionnaires), and serum concentrations of gastrointestinal hormones (assessed via enzyme-linked immunosorbent assay). ML can efficiently classify pediatric IFC into its three subtypes. Notably, among the models tested, the LGBM model proved to be the most accurate, achieving a high accuracy of 83.8%, demonstrating the effectiveness of machine learning as a tool for managing IFC in children.

The manuscripts published in this special issue, including reviews of original articles and methods covering different clinical conditions, would be beneficial to professional knowledge and clinical care for this population.

